# Chitosan-Al₂O₃ green hydrogel composites: a sustainable approach for Cr(VI) removal from simulated solution

**DOI:** 10.1186/s13065-025-01633-9

**Published:** 2025-10-06

**Authors:** Rasha S. Kamal, Ebtehal Mosaad Ahmed, Amr Feteha, Mohamed S. Behalo, Manar E. Abdel-Raouf

**Affiliations:** 1 Petroleum Application Department, Egyptian Petroleum Research Institute, Nasr City, Cairo, 11727 Egypt; 2 Organic Chemistry Laboratory, Chemistry Department, Faculty of Science, Benha University, P.O. Box 13518, Benha, Egypt

**Keywords:** Chitosan, Carboxymethyl chitosan, Cr(VI), Kinetic models, Kinetic isotherms, AFM

## Abstract

Extensive industrial activities discharge huge amounts of different pollutants into water bodies. Among these pollutants, heavy metals stand as the most poisonous species due to their resistance to biodegradation and their short- and long-term exposure effects. More specific, Cr(VI) is one of the top-five toxic elements that pose potent toxicity to the entire environmental system. In this study, six modified chitosan hydrogel composites (Categorized in two groups and comprised of chitosan or carboxymethyl chitosan crosslinked with acrylamide and incorporating aluminum oxide as an inorganic core) were prepared under the influence of gamma irradiation at an optimized dose (30 kGy) as a facile, environmentally friendly technique. These hydrogels were employed for the removal of Cr(VI) from aqueous solution considering various variables that influencing removal performance, taking structural variation into consideration. The removal process was followed by the AFM to compare between the chromium-free and chromium-loaded surfaces. According to the experimental findings, the following circumstances were ideal for maximizing dye uptake by the optimized samples: pH 2, 120-min contact duration, 0.1 g of sorbent, and a metal concentration of 50 ppm. The maximum metal uptake was achieved by the prepared green sorbents was found competitive (ranging from 48.9 to 51.5 mg/g). Moreover, all the investigated sorbents showed strong removal efficiency and adsorption capability after four cycles of sorption/desorption. However, it was found that the adsorption capacities of the second group's elements was higher than that of the first group. Additionally, the data demonstrated that the adsorption process conformed the pseudo-first order kinetic isotherm and best fit the Freundlich model imposing multilayer adsorption of Cr(VI) onto the sorbent matrix via several mechanisms which is consistent with variable functionalities in the hydrogel matrix.

## Highlights

- Six crosslinked chitosan composites were produced using gamma irradiation at a dosage of 30 kGy.
- To remove Cr(VI) from aquatic settings, green sorbents were used.
- It was confirmed that the structural variation and metal removal performance were correlated.
- The AFM was used to track the removal procedure.

**Graphical abstract:**

## Graphical abstract

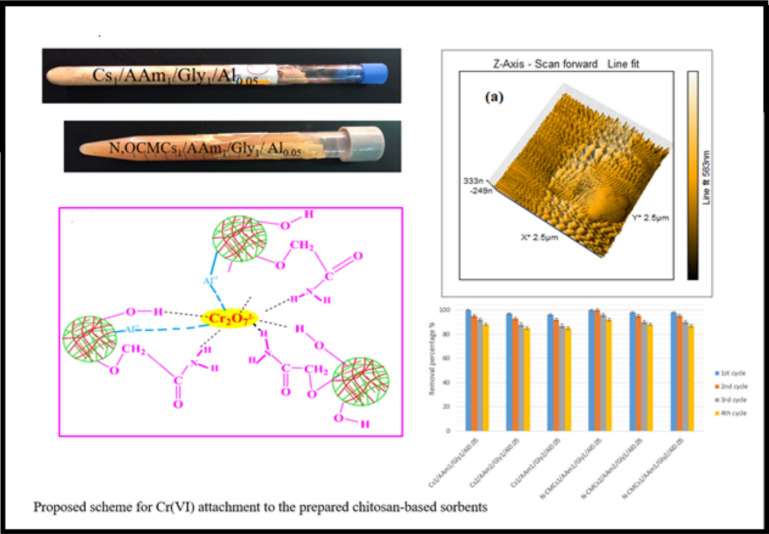

## Introduction

Pollution is a key contributor to water scarcity, exacerbated by urbanization and industrialization. With global waste generation rising rapidly, sludge output is projected to reach 2–3 billion metric tons annually by 2050. Water pollution stems from both natural and human factors, with human actions being the dominant cause. Pollutants are categorized into inorganic (e.g., heavy metals, radioactive waste), microbiological (e.g., viruses, bacteria), and organic (e.g., pharmaceuticals, insecticides). The increasing complexity of water pollution underscores the urgent need for sustainable water management and pollution control [7, 8, 34], Nahlah [28, 40, 44, 46, 58, 72]. Environmental buildup of inorganic pollutants can pose serious health hazards to ecosystems and people alike. Arsenic, cadmium, chromium (VI), lead and mercury are the top-five heavy metals with potent hazardous effects due to their carcinogenic, bio accumulative, and non-biodegradable nature [64, 70, 72]. The concentrations of these specific metals in drinking water are strictly regulated by health organizations like the WHO. They are discharged into water bodies as effluents of different industries such as mining, smelting, battery manufacturing, dye production, and electronics. To mitigate this, various removal techniques are employed, including chemical precipitation, oxidation, reverse osmosis, electrodialysis, and adsorption [3, 5, 11, 13, 25, 26, 35, 39, 45, 55, 65, 69].

Among these, adsorption is particularly effective, offering a cost-efficient and versatile solution for removing heavy metals from wastewater [30]. Sorbents for pollutant removal can be fall into two primary groups: natural and synthetic [1]. Natural adsorbents, such as clay minerals, zeolite, charcoal, rice husks, fly ash, sawdust, coconut shells, and seafood processing wastes are abundant, cost-effective, and adaptable for improved adsorption performance [33, 74]. Recent advancements emphasize developing biodegradable, cost-effective, and eco-friendly adsorbents from modification of natural polymers. In this regard, chitosan (a deacetylated chitin) stands as the second abundant natural polymer with high functionalities due to its –NH₂ and –OH groups, which enable various modification protocols to enhance its adsorption performance such as esterification, carboxymethylation and crosslinking [18, 20, 32, 33, 36, 41, 49–51, 53, 54, 61]. Chemically, chitosan is a typical polysaccharide made of N-acetyl-D-glucosamine connected by β-1,4-glycoside linkages [19]. Environmentally, chitosan and its derivatives are highly effective in wastewater treatment, a topic explored in detail in subsequent sections 29, 62].

On the other hand, high levels of toxic chromium are produced by different industries, particularly in its hazardous hexavalent form, Cr(VI) which can penetrate cell membranes and oxidize biological molecules. According to WHO guidelines, Cr(VI) levels in drinking water should not exceed 0.050 mg/L [71]. Therefore, reduction of Cr(VI) level below this concentration is very critical. In this regard, Several chitosan based composites were applied for the removal of Cr(VI) [1]. In addition, supporting hydrogel matrices with inorganic species such as clay [43], biochar [9] and metal oxides [66] enhances the mechanical properties, increases number of the reusability cycles and overall improves the workability of the composite materials [4, 27, 56, 57].

Following our interest in harnessing natural polymer in environmental remediation, the present work concerns synthesis of two sets of hydrogel materials: one made up from raw chitosan while the other comprises of carboxymethyl chitosan. In the two groups, Al_2_O_3_ was used to strengthen the hydrogels in order to maintain their consistency and enhance their general qualities. More interestingly, the crosslinked hydrogels were constructed using an optimized dose of gamma irradiation (at 30 kGy) as per our previous work [4].

Gamma- induced crosslinking established superior energy efficiency when compared to thermal methods, requiring ~ 30% less energy input by abolishing high-temperature processing. Gamma irradiation also enhanced structural consistency and pore relative uniformity. Additionally, gamma processing guarantees faster processing times (minutes vs. hours), deeper penetration for bulk materials, and elimination of toxic chemical initiators—critical for sustainable biomaterial production [27, 42].

In the first part of this work, the preparation methodologies were clearly demonstrated and the prepared hydrogels were thoroughly described. In order to establish the concepts of sustainability and the bases of circular economy, the aforementioned hydrogels were employed for the removal of Cr(VI) from simulated solution. Various application settings were applied in order to attain optimized conditions. Afterwards, the hydrogels were used to remediate a sample an industrial wastewater. At last, the adsorption process was comprehensively investigated by different kinetic models and isotherms to understand the nature of the overall process. The adsorption process was also verified through and AFM investigation for hydrogels before and after metal adsorption [4].

## Materials and methods

### Materials

Low-molecular-weight chitosan (Cs) was provided from Sigma-Aldrich (Germany) as yellowish white fine powder with a 75–80% degree of deacetylation. Acrylamide (AAm) (of purity 99.9%) was purchased from Aldrich in Germany. The additional reagents such as potassium dichromate (K₂Cr₂O₇), aluminum oxide (Al_2_O_3_), chloroacetic acid, Glacial acetic acid, hydrochloric acid, and analytical-grade sodium hydroxide (NaOH) were obtained from El-Nasr Co. (Cairo, Egypt) Distilled water was used throughout the experimental work.

### Carboxymethylation of chitosan (N,O-CMCs) [4]

The detailed methodology was mentioned in our previous work. Carboxymethylation reaction of chitosan into carboxymethyl chitosan (N,O-CMCs) was conducted in a strong alkaline condition. Shortly, 5 g of chitosan powder was suspended in 90 mL of isopropanol while being constantly stirred at 350 rpm. Then, 18.4 g of monochloroacetic acid was suspended in 90 mL isopropanol and 24.4 g of aqueous NaOH (40%) were then added. The reaction was continued at 40 °C for an hour then at room temperature for three hours. The final product was neutralized with glacial acetic acid after being filtered and suspended in 150 mL of methanol. The product was dried in an open-air oven at 50 °C after being repeatedly cleaned with 80% ethanol.

### Preparation of crosslinked composite hydrogels

The crosslinked hydrogels were created by dissolving the desired quantities of Cs or N,O-CMCs in 100 mL of 5% acetic acid solution and the mixture was sonicated for 30 min to achieve homogeneity. Next, the specified quantity of AAm was dissolved in 10 mL of DW and added to the previous solution and subjected to further sonication for 30 min. According to the composition mentioned in our previous work [4], glycerol was added to improve flexibility. Before subjected to gamma irradiation to induce crosslinking reaction, 0.05 g of Al_2_O_3_was dissolved in 10 mL of distilled water, added to the reaction mixture and continuously stirred for two hours at 50–60 °C. The final composition was poured into Pyrex glass tubes and exposed to 30 kGy of gamma radiation. The hydrogel rods were sliced into uniform discs, air-dried, and further purified by extraction in distilled water at 70 °C for 2 h as shown in Table 1.

**Table 1:** The codes and composition of the green sorbents [4]

Code/composition	Cs	AAm	Al_2_O_3_	Gly
*Group*	Group A*			
Cs_1_/Aam_1_/Gly_1_/Al_0.05_	0.17	0.17	0.05	0.17
Cs_2_/AAm_2_/Gly_1_/Al_0.05_	0.34	0.34	0.05	0.17
Cs_1_/AAm_1_/Gly_2_/Al_0.05_	0.17	0.17	0.05	0.34
Group B*				
N,O-CMCs_1_/AAm_1_/Gly_1_/Al_0.05_	0.17	0.17	0.05	0.17
N,O-CMCs_2_/AAm_2_/Gly_1_/Al_0.05_	0.34	0.34	0.05	0.17
N,O-CMCs_1_/AAm_1_/Gly_2_/Al_0.05_	0.17	0.17	0.05	0.34

*The reactants are added in grams

### Characterization of composites [4]

The synthesized composites were analyzed using various techniques:

- Functional group Identification was performed through FTIR spectrophotometer model Perkin-Elmer 720 in the range (400–4000 cm⁻1).
- Morphological Studies: Scanning electron microscopy (JEOL GSM 6510LV) was employed morphological features.
- The topographic mappings of the hydrogels after adsorption were investigated by atomic force microscopy (AFM Flexaxiom, C3000) in non-contact mode using silicon cantilever
- Crystallinity Analysis: X-ray diffraction (Shimadzu diffractometer, Cu Kα radiation, 2θ range: 10°–80°) was used to confirm the crystallinity of the composites.
- Thermal Analysis: Thermogravimetric analysis (Q600 SDT, 30–800 °C, 10 °C/min under N₂) was conducted to verify the thermal properties of the hydrogel composites.
- Surface Area Analysis: BET sorptometry (BET-Belsorp-Minix, Japan) was used following degassing at 120 °C for 9 h to assess the BET properties of the hydrogels.
- UV–Vis Spectroscopy: The removal performance was calculated by the aid of a double beam UV/Vis spectrophotometer, model Peak 702 (at wave length for Dichromate ion (Cr_2_O_7_^2−^ in acidic medium: λ_max_ = 350 nm (strong absorption) and Chromate ion (CrO_4_^2−^​) in basic/neutral medium: λ_max_ = 372 nm).

### Adsorption studies

A stock solution of Cr(VI) [500 ppm of K_2_Cr_2_O_7_] was prepared and used for adsorption experiments by a tea-bag method at different conditions (0.1 g of sorbent material).

- Effect of pH: 2, 4, 6, 8 and 10.
- Effect of temperature: 30, 40 and 50 °C
- Effect of Cr(VI) concentration: 25, 50, 75 and 100 ppm.
- Effect of contact time: tell reaching plateau

The following formulas were used to get the adsorption capacity (q) and % removal: where V is the solution volume (L), W is the adsorbent mass (g), and C_₀_, C_t_, and C_e_ are the initial, time-dependent, and equilibrium concentrations (mg/L), respectively.

1 $${\text{q}}_{t}=\frac{{(C}_{0}-{C}_{t}) V}{W}$$

2 $${\text{q}}_{e}=\frac{{(C}_{0}-{C}_{e}) V}{W}$$

3 $$\text{The removal }\left(\text{\%}\right)=\frac{{C}_{0}-{C}_{f}}{{C}_{0}}\times 100$$

### Kinetic and isotherm models

Adsorption kinetics were assessed using pseudo-first-order and pseudo-second-order models:

4 $${{\text{log}(\text{q}}_{\text{e}}-\text{q}}_{\text{t}})={\text{log q}}_{\text{e}}-\frac{{\text{K}}_{1}}{2.303}\text{t}$$

5 $$\frac{t}{{\text{q}}_{t}}=\frac{1}{{k}_{2}{q}_{e}^{2}}+\frac{t}{{\text{q}}_{e}}$$

where the pseudo-first- and pseudo-second-order rate constants are denoted by k₁ and k₂, respectively. The amounts of Cr(VI) adsorbed at equilibrium (in mg/g) and at a certain time t (in mg/g) are denoted by the variables q_e_ and q_t_, respectively.

To simulate adsorption isotherms, the Langmuir and Freundlich equations were utilized:

6 $$\frac{{\text{C}}_{\text{e}}}{{\text{q}}_{\text{e}}}= \frac{1}{({\text{q}}_{\text{max}}. {\text{K}}_{\text{L}})}+\frac{{\text{C}}_{\text{e}}}{{(\text{q}}_{\text{max}})}$$

7 $$\text{Ln}{ q}_{e }=\text{ln}{K}_{F}+\frac{1}{n}\text{ln}{C}_{e}$$

where q_e_ (mg/g) is the adsorbed amount at equilibrium and Ce (mg/ L) is the metal's equilibrium concentration at a certain temperature. constants KF and n that are related to the adsorption's intensity and capacity, qₘ and Kₜ indicate the Langmuir parameters, and Kᴷ, n denote the Freundlich constants.

### Regeneration studies

Spent hydrogels were regenerated by immersion in 25 mL of an alkaline solution (0.1N NaOH), followed by washing with distilled water to neutral pH. Hydrogels were dried at 50 °C before reuse. Adsorption efficiency was assessed over four cycles to determine regeneration performance.

### Remediation of a real industrial wastewater sample

The wastewater samples were collected on private land in the 10th of Ramadan City. The adsorption via tea bag method was applied onto the optimized samples on a real textile wastewater sample with initial composition (Cu^+2^: 131 ppm, Cr^+6^: 33.8 ppm, Hg^+2^ = 21.17 ppm and Pb^+2^ = 14.18 ppm) after two hours at the optimized conditions.

## Results and discussion

### Gel formation and structure verification

Chitosan, carboxymethyl chitosan (CMCs), and acrylamide were mixed in different mass ratios for constructing hydrogel composites through gamma irradiation, forming a crosslinked structure with reactive functionalities. Aluminum ions (Al^3^⁺) were incorporated into the polymer matrix through electrostatic attraction, enhancing the thermal stability of the hydrogel and creating active centers for adsorption. Glycerol, used as a plasticizer, improved flexibility but did not participate in crosslinking. Fourier-transform infrared spectroscopy (FTIR) inveterate the presence of key functional groups and confirmed crosslinking. The gel fraction analysis showed that higher glycerol content resulted in increased gelation, supporting its role in modulating hydrogel properties. X-ray diffraction (XRD) patterns indicated the presence of Al₂O₃ and confirmed the amorphous nature of the hydrogels. Thermal analysis using TGA/DTA demonstrated that the hydrogel composites were thermally stable, with N,O-CMCs-based hydrogels exhibiting superior stability due to increased crosslinking. Scanning electron microscopy (SEM) revealed a porous morphology in N,O-CMCs-based composites, favoring dye adsorption, while chitosan-based composites had a denser, layered structure. Atomic force microscopy (AFM) confirmed variations in surface roughness and height, correlating with acrylamide and glycerol content. BET surface area analysis indicated that N,O-CMCs-based hydrogels had higher porosity and surface area, making them more effective for adsorption. Antimicrobial activity tests against E. coli and S. aureus showed that chitosan-based composites exhibited stronger antibacterial effects due to their higher amine content. These findings demonstrate the potential of these hydrogels for applications in adsorption and antimicrobial treatments [4]. These data were published in the previous work [4].

### BET data for the prepared hydrogels

The internal features of the prepared hydrogels were accessed via BET (Brunauer–Emmett–Teller) by using N_2_ adsorption–desorption isotherm graphs. This method affords critical insights into the surface area and porosity of hydrogel materials, which are key factors influencing their adsorption performance. A higher BET surface area typically indicates more available active sites for adsorption, potentially enhancing the hydrogel's capacity to extract Cr(VI) from water-based solutions. Table 2 lists the BET characteristics, which include surface area, average pore volume (Dubinin-Astakhov), and average pore diameter. It is evident that the N,O-carboxymethyl chitosan-based groups outperformed the chitosan-based ones in terms of average pore volume and total surface area. Furthermore, the first group's elements are microporous, whereas the second group's elements are mesoporous. These findings are very important to interpret the variation in adsorption capacities of the prepared hydrogels with respect to their constituent composition. Tekay et al. studied chitosan/montmorillonite composite hydrogels incorporating Spirulina biomass with a BET surface area of 1.51 m^2^/g. Despite this relatively low surface area, the composite hydrogel achieved a maximum Cr(VI) adsorption capacity of 178 mg/g. This suggests that factors beyond surface area, such as the presence of functional groups and the hydrogel's structural properties, significantly contribute to adsorption efficiency [67]. ​Pavithra et al. investigated the removal performance of chitosan/orange peel hydrogel towards some heavy metals including Cr(VI) and Cu(II). The data showed that the inclusion of orange peel enhanced the hydrogel's porosity and surface area, leading to improved adsorption capacities for Cr(VI) ions ​[48]. Overall, it's important to note that while a higher BET surface area generally correlates with better adsorption performance, other factors such as pore size distribution, functional group availability, and the chemical nature of the hydrogel also play significant roles. Therefore, a comprehensive assessment of adsorption performance should consider these factors alongside BET surface area measurements.

**Table 2:** BET data of the investigated sorbents

Sorbent	Total surface area (m^2^/g)	Average pore volume (cc/g)	Average pore diameter (nm)
Cs1/AAm1/Gly1/Al0.05	19.63	0.016	1.92
Cs2/AAm2/Gly1/Al0.05	12.14	0.031	1.14
Cs1/AAm1/Gly2/Al0.05	11.41	0.012	1.05
N,O-CMCs1/AAm1/Gly1/Al0.05	21.41	0.349	6.43
N,O-CMCs2/AAm2/Gly1/Al0.05	16.22	0.217	5.76
N,O-CMCs1/AAm1/Gly2/Al0.05	15.87	0.208	4.65

### AFM characterization of Cr(VI) loaded chitosan-Al_2_O_3_ composites

**Fig. 1:**
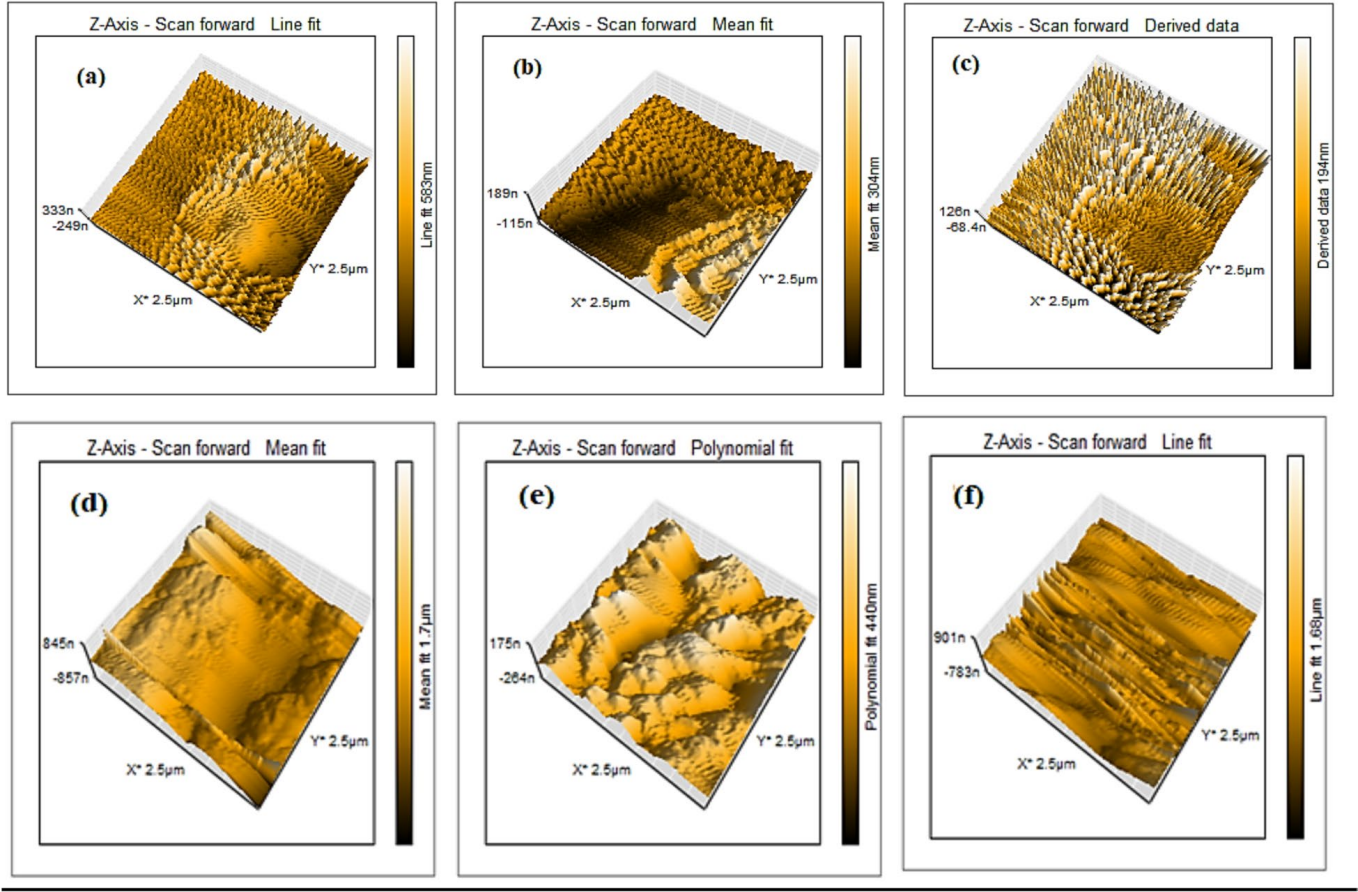
AFM images of Cr(VI) loaded composites, **a** Cs1/AAm1/Gly1/Al0.05, **b** Cs2/AAm2/Gly1/Al0.05, **c** Cs1/AAm1/Gly2/Al0.05, **d** N,O-CMCs1/AAm1/Gly1/Al0.05, **e** N,O-CMCs2/AAm2/Gly1/Al0.05 and **f** N,O-CMCs1/AAm1/Gly2/Al0.05

The AFM images of the metal loaded composites are given in Fig. 1. It can be seen that there is an obvious variation in the height measurements and surface features reflecting the metal loading capacities of the hydrogel. More interestingly, the data agree with the BET findings which reveal better adsorption performance for the composites of the second group comprising modified chitosan than the first group containing chitosan. It is assumed that the increased functionalization enhances the binding sites for metal binding. In addition, the increased monomer feed ratio may induce more crosslinking which negatively affects the adsorption due to blocking of fine pores responsible for better entrapping of the adsorbed species. Therefore, the order of adsorption performance is as following: N,O-CMCs1/AAm1/Gly1/Al0.05 > N,O-CMCs2/AAm2/Gly1/Al0.05 > N,O-CMCs1/AAm1/Gly2/Al0.05 and in the same order for the elements of the first group.

### The removal of Cr(VI) from simulated solutions

The prepared nanocomposites were employed for The removal of Cr(VI) under various application circumstances, such as temperature, pH, contact duration, starting Cr(VI) content, and composite weight.

#### Effect of pH

**Fig. 2:**
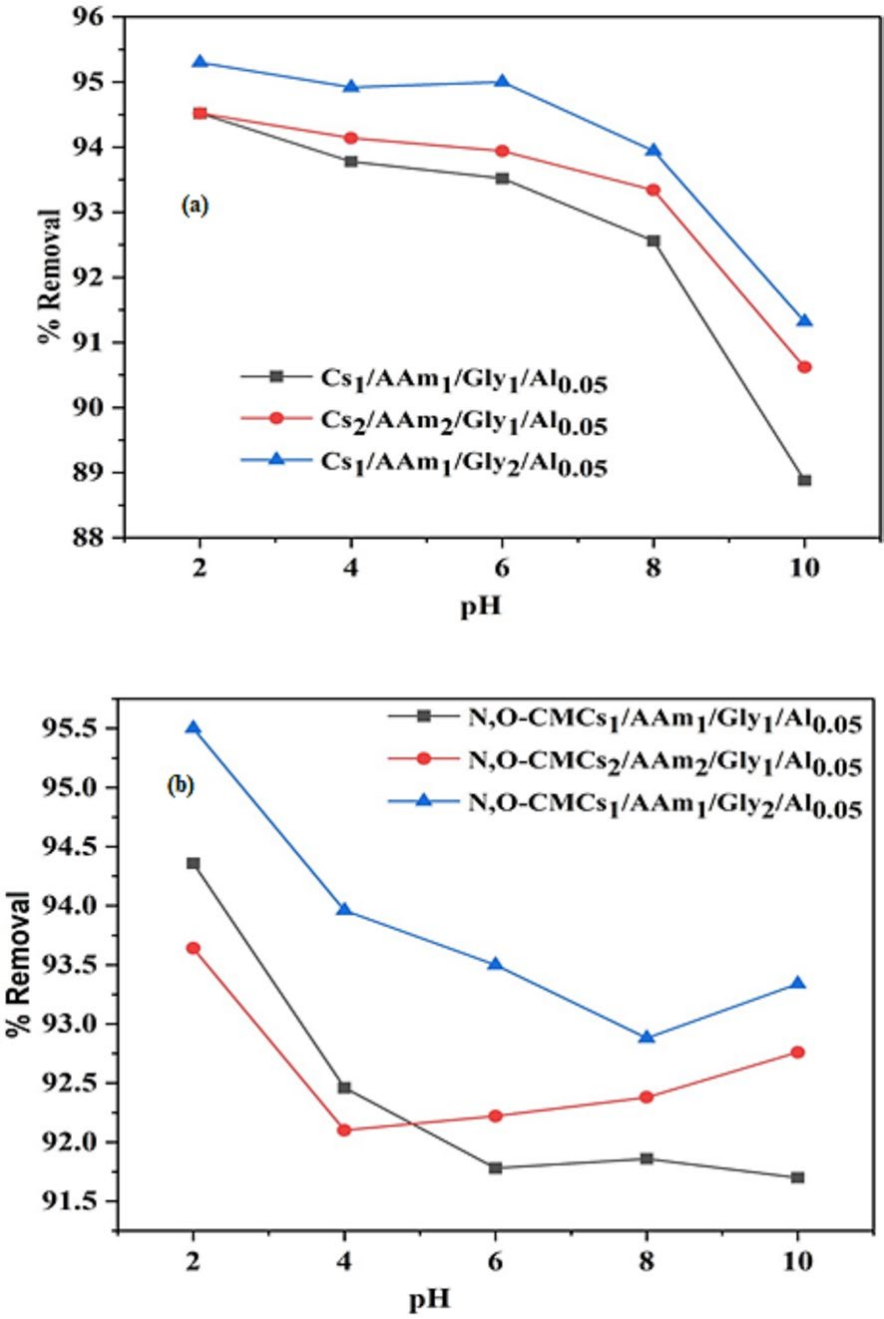
Effect of pH on % removal of Cr (VI) onto **a** Cs_1_/AAm_1_/Gly_1_/Al_0.05,_ Cs_2_/AAm_2_/Gly_1_/Al_0.05,_ Cs_1_/AAm_1_/Gly_2_/Al_0.05_, **b** N,O-CMCs_1_/AAm_1_/Gly_1_/Al_0.05,_ N,O-CMCs_2_/AAm_2_/Gly_1_/Al_0.05,_ N,O-CMCs_1_/AAm_1_/Gly_2_/Al_0.05_ hydrogels (RT, 0.1 g sorbent, 50 ppm Cr(VI))

The pH of the solution significantly affects the removal performance of chitosan-based composites for Cr(VI) removal due to changes in surface charge, Cr(VI) speciation, and adsorption mechanisms. The pH- Cr(VI) removal relationship for group A (Cs_1_/AAm_1_/Gly_1_/Al_0.05,_ Cs_2_/AAm_2_/Gly_1_/Al_0.05,_ Cs_1_/AAm_1_/Gly_2_/Al_0.05_) and group B (N,O-CMCs_1_/AAm_1_/Gly_1_/Al_0.05,_ N,O-CMCs_2_/AAm_2_/Gly_1_/Al_0.05,_ N,O-CMCs_1_/AAm_1_/Gly_2_/Al_0.05_) was presented in Figs. 2a and 1b respectively. The investigation of the effect of pH was carried out over a range of pH 2–10 under the following circumstances: 100 ml of aqueous solution, 0.1 g of adsorbent, 30 min of contact time, and an initial concentration of 50 ppm of Cr (VI). The findings showed that the maximum removal performance for all the investigated hydrogels was attained at pH 2. This finding is attributed to the protonation and ionization of the functional groups of the composites allowing better interaction with Cr(VI) ions. By increasing the pH of the solution, the removal performance significantly decreased [14, 38, 59]. This behavior is explained as following:

a Effect of pH on type of Cr(VI):
  At low pH (< 3): Cr(VI) exists chiefly as HCrO₄⁻ and some Cr₂O₇^2−^.
  At neutral to alkaline pH (> 6): Cr(VI) is present mostly as CrO₄^2−^.
b Adsorption Mechanism at different pH:
  At low pH (1–3): Maximum adsorption occurs because chitosan is highly protonated to form (NH_3_^+^) enhancing electrostatic attraction with negatively charged Cr(VI) species (HCrO₄⁻, Cr₂O₇^2−^).
  At neutral pH (5–7): Adsorption efficiency decreases as chitosan deprotonates, reducing electrostatic attraction.
  At high pH (> 8): Poor removal efficiency due to repulsion between negatively charged CrO₄^2^⁻ and deprotonated chitosan surface. In conclusion, the Optimal pH for Cr(VI) Removal is achieved in the acidic range (pH 2–4) due to strong electrostatic interactions. At pH > 6 a reduced efficiency was observed as Cr(VI) exists mainly as CrO₄^2^⁻, which is less attracted to chitosan. Our data runs parallel to those mentioned in some recent studies which have demonstrated that the pH of a solution significantly influences the efficiency of chitosan-based composites in removing hexavalent chromium [Cr(VI)] from aqueous environments. Generally, the composites prepared in that work (two crosslinked modified chitosan biopolymers, CTS-VAN and Fe₃O₄@CTS-VAN) exhibited higher adsorption capacities under acidic conditions, with optimal performance typically observed around pH 3 to 4. The X-ray photoelectron spectroscopy (XPS) analysis revealed that Cr(III) accounted for 83% of the total chromium bound to the bioadsorbents' surface, suggesting that reductive adsorption played a key role in Cr(VI) removal [37].

#### Effect of contact time

**Fig. 3:**
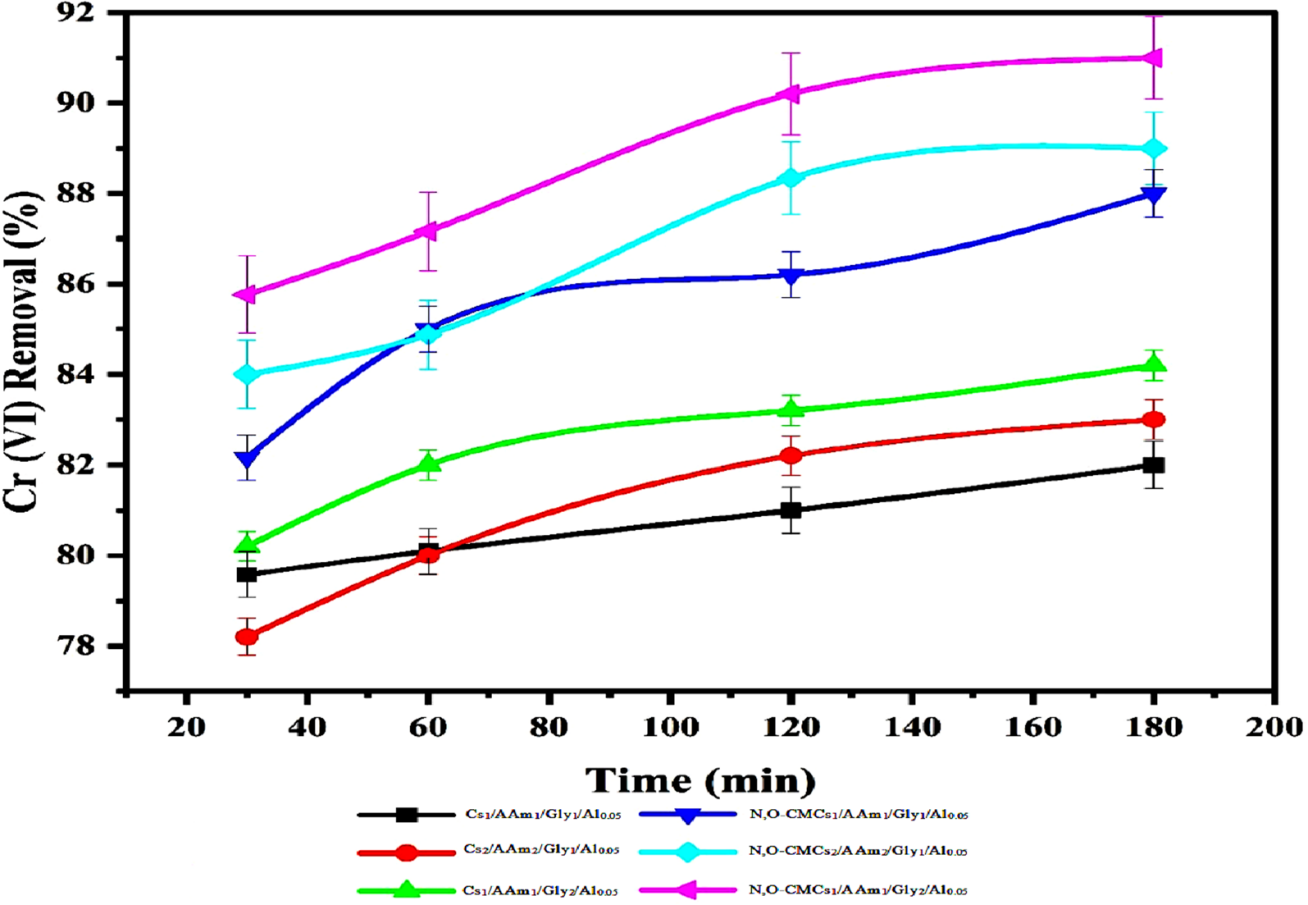
Collective graph showing the effect of contact time on the Cr (VI) removal % onto the prepared hydrogels at optimum conditions (Rt, 50 ppm of Cr(VI), 0.1 g sorbent, pH = 2)

The contact time-rate of adsorption relationship between chitosan-based composites and hexavalent chromium [Cr(VI)] is given in Fig. 3. It can be seen that the duration of contacting between the sorbent material and the anionic species significantly influences the removal efficiency by exhibiting a biphasic profile as following: ​

- Swift Initial Adsorption: In the initial phases, there is an immediate uptake of Cr(VI) due to the abundance of available active sites on the adsorbent's surface [77].​
- Deliberate Equilibration Phase: As the active sites become occupied, the adsorption rate declines until reaching equilibrium [16]. ​

Thus, a considerable amount of Cr(VI) was initially adsorbed onto the active sites present in the sorbent matrix in the first two hours, indicating an instant adsorption process and that the rate of adsorption was very fast due to the strong interactions between Cr(VI) groups and the nanocomposite hydrogel. After that, the majority of accessible binding sites being occupied, the adsorption rate reduced after achieving equilibrium [27, 31]. Our data are consistent with some findings for other chitosan-based sorbents. For instance, a study on chitosan-silicon-hydroxyapatite (Cs–Si–Hap) composites observed that approximately 91% of Cr(VI) removal occurred within the first 10 min, with equilibrium achieved at 10 min. In contrast, chitosan alone reached equilibrium at 30 min, highlighting the enhanced performance due to composite formation [16]. The same pH value was noticed by Naicker et al. for maximum adsorption of Cr(VI) onto Magnetic Chitosan/Graphene Oxide/Metal Oxide Composite Beads. These beads exhibited maximum Cr(VI) adsorption capacities of approximately 78 mg/g at pH 2 and 298 K [47].

Similarly, research involving a hybrid membrane of carboxymethyl chitosan and silicon dioxide demonstrated that the adsorption capacity increased with time, achieving about 80% removal efficiency at 60 min, after which no significant increase was observed [22]. These observations suggest that while the majority of Cr(VI) removal by chitosan-based composites occurs rapidly, the system requires a specific duration to attain equilibrium, with the exact time frame varying based on the composite's composition and structural properties.

#### Effect of temperature

**Fig. 4:**
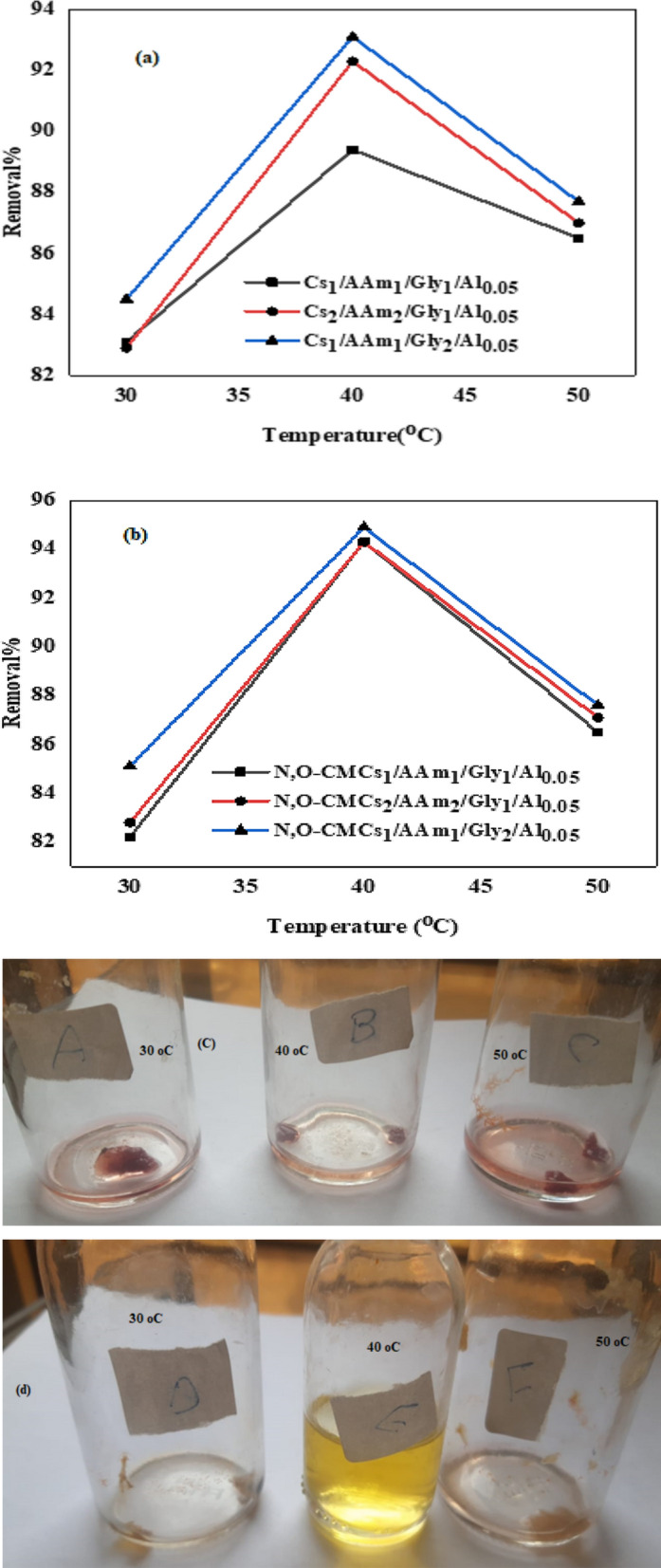
Effect of temperature on the Cr (VI) % removal % of **a** Cs_1_/AAm_1_/Gly_1_/Al_0.05_, **b **Cs_2_/AAm_2_/Gly_1_/Al_0.05,_ **c** Cs_1_/AAm_1_/Gly_2_/Al_0.05_ **c** Cs_1_/AAm_1_/Gly_1_/Al_0.05_ and **d** N,O-CMCs_1_/AAm_1_/Gly_1_/Al_0.05_ at optimum conditions

One of the most crucial factors that can enhance or inhibit the adsorption process is the temperature of the medium due to its direct effect on a number of variables, including the stability of the chelate structure, the mobility of metal cations or ionic groups, and the dilatation or shrinkage of pores [15]. The effect of temperature on adsorption of Cr(VI) onto chitosan sorbents is displayed in Fig. 4a and b for group A and B respectively. In addition, camera images for the effect of temperature on removal performance of Cs_1_/AAm_1_/Gly_1_/Al_0.05_ and N,O-CMCs_1_/AAm_1_/Gly_1_/Al_0.05_ are given in Fig. 4c and d respectively. The experimental findings show that the initial removal process in endothermic in nature and was enhanced and reached maximum by changing the temperature from 30º to 40° then it decreased at further increase from 40° to 50 °C. This may be attributed to the enhancement in the kinetic energy of the travelling species by moderate rise of temperature. At higher temperature, the polymer matrix may be squeezed or shrinked leading to desorption of the adsorbed entities. The same behavior was noticed on the removal of Cr(VI) by Cassava peels carbon [15]. For instance, a study on chitosan-based hydrogels demonstrated that higher temperatures improved Cr(VI) removal efficiency, suggesting that elevated temperatures facilitate the adsorption process. Thermodynamic analyses across various studies consistently reveal that the adsorption of Cr(VI) onto chitosan-based hydrogels is spontaneous and endothermic, with positive enthalpy (ΔH°) values indicating that higher temperatures favor the adsorption process. These findings collectively suggest that increasing the temperature enhances the removal performance of chitosan-based hydrogels for Cr(VI), likely due to increased kinetic energy facilitating more effective interactions between Cr(VI) ions and the hydrogel's active sites. However, it is suggested that at certain temperature, a two- direction process including sorption/desorption may occur due to the occupation of active sites due to enhanced kinetic energy of the travelling species and then a competition between the sorbed and drifting species inhibit the adsorption process [17, 24].

#### Effect of metal ion concentration

**Fig. 5:**
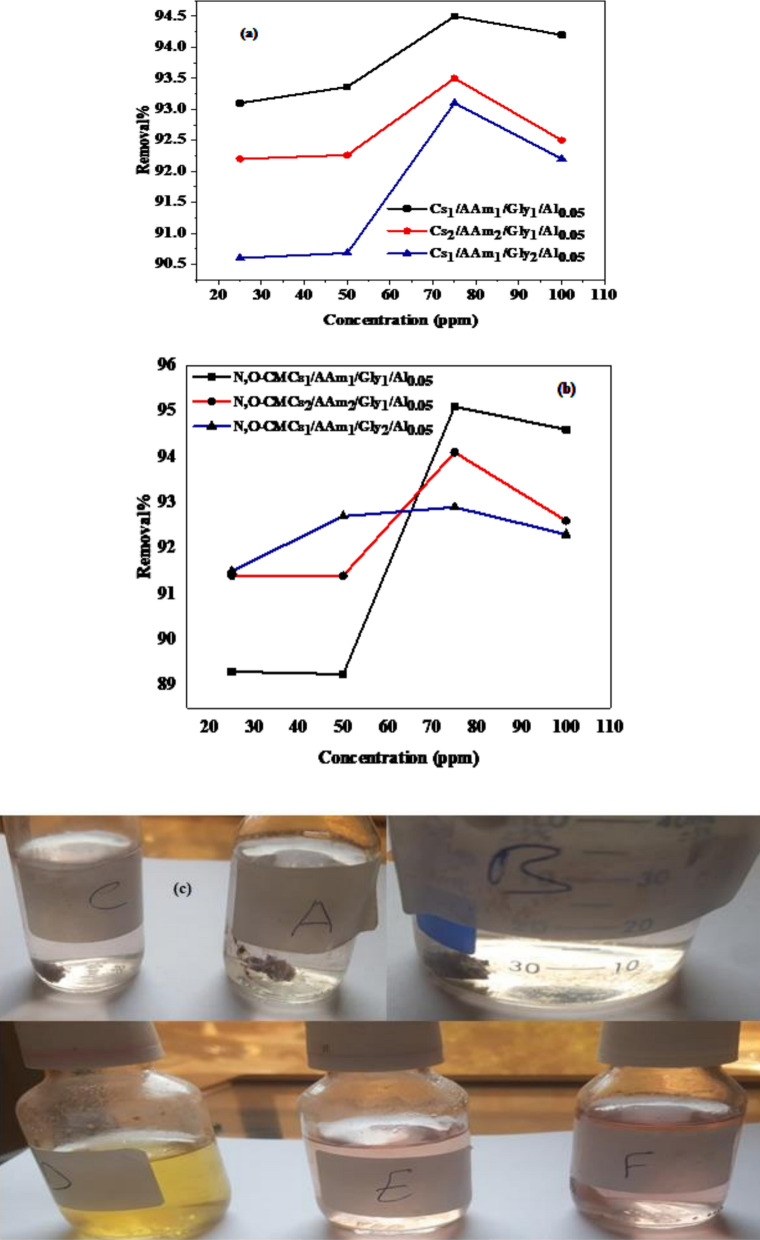
Effect of initial metal ion concentration on the Cr (VI) removal % of **a** Cs_1_/AAm_1_/Gly_1_/Al_0.05,_ Cs_2_/AAm_2_/Gly_1_/Al_0.05,_ Cs_1_/AAm_1_/Gly_2_/Al_0.05_, **b** N,O-CMCs_1_/AAm_1_/Gly_1_/Al_0.05,_ N,O-CMCs_2_/AAm_2_/Gly_1_/Al_0.05,_ N,O-CMCs_1_/AAm_1_/Gly_2_/Al_0.05_ hydrogels at optimum conditions and **c** Illustration of maximum removal at the optimized conditions on A = Cs_1_/AAm_1_/Gly_1_/Al_0.05_, B = Cs_2_/AAm_2_/Gly_1_/Al_0.05,_ C = Cs_1_/AAm_1_/Gly_2_/Al_0.05,_ D = N,O-CMCs_1_/AAm_1_/Gly_1_/Al_0.05,_ E = N,O-CMCs_2_/AAm_2_/Gly_1_/Al_0.05_ and F = N,O-CMCs_1_/AAm_1_/Gly_2_/Al_0.05_

Initial Metal ion concentration is another factor that governs removal performance process in respect to the unoccupied active sites in the polymer matrix. At low concentrations, the removal process was very rapid and instant due to the abundancy of active sites. However, at a certain concentration where the active sites are all occupied by the adsorbed entities, the removal process was hindered and the rate of adsorption equals to the rate of desorption and then a stability state was attained. This behavior is illustrated in (Fig. 5a) and (Fig. 5b) for groups A and B respectively. It can be seen that the removal percentage increases by increasing the Cr(VI) concentration up to 80 ppm. Beyond this concentration, a drastic depletion was observed. One can deduce that the maximum removal percentage achieved by the elements of both groups is 80 ppm. An illustration of the maximum removal performance attained by the prepared sorbents at the optimized experimental conditions is given in (Fig. 5c). Our data is very comparable to Abdel-Wahab et al. in their study on a quinoxaline chitosan Schiff base (CsQ) where enhanced adsorption was noticed at low initial Cr(VI) concentrations (5 to 50 ppm), and the removal efficiency reached a maximum at 50 ppm. Beyond this concentration, the removal efficiency declined, dropping from 36.9 to 24.4% as the concentration increased from 50 to 200 ppm. This decrease is attributed to the saturation of active sites on the polymer, limiting further adsorption [2]. Similarly, an exploration on chitosan hydrogel beads crosslinked by Cu^2+^ ions (CS-Cu-B) verified that the adsorption capacity for Cr(VI) increased with higher initial Cr(VI) concentrations, indicating that the hydrogel's active sites were effectively utilized up to a certain point. However, the study also noted that beyond an optimal concentration, the adsorption capacity plateaued, suggesting saturation of the available binding sites [73]. These findings indicate that while increasing the initial Cr(VI) concentration can enhance the adsorption capacity of chitosan-based hydrogels up to a certain threshold, exceeding this optimal concentration leads to a decline in removal efficiency due to the saturation of active sites [10, 63].

### Reusability of the prepared sorbents towards Cr(VI) after repeated adsorption/desorption cycles

**Fig. 6:**
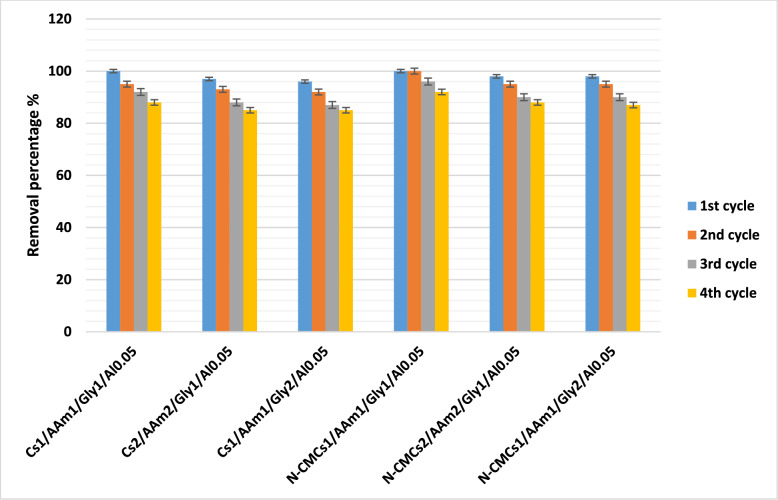
Reusability investigation of the prepared sorbents towards Cr (VI)

The removal performance of the prepared hydrogels towards Cr(VI) was investigated for four successive sorption/desorption cycles, (Fig. 6). It can be seen that the removal performance slightly decreased by repeated usage. This finding assures the suitability of these hydrogel composites in wastewater treatment application [21]. Combining the data of removal of MR dye [4]. one can say that these multi-functional sorbents are excellent candidates in removing different dyes from polluted water [73]. The reusability of chitosan-based hydrogels for Cr(VI) removal is a critical factor for their practical application in wastewater treatment. Effective reusability implies that the hydrogel can maintain its adsorption capacity over multiple adsorption–desorption cycles, thereby reducing operational costs and waste generation. In this regard, several studies have demonstrated the favorable reusability of these hydrogels: ​

- Chitosan Hydrogel Beads Crosslinked by Cu^2^⁺ Ions (CS–Cu–B) shown exceptional reusability by maintaining 87.90% of its adsorption capacity after five adsorption–desorption cycles and demonstrating a maximum Cr(VI) adsorption capacity of 90.76 mg/g at pH 4.0.
- Even after four regeneration cycles, the Chitosan/Cellulose Nanocrystals/Carbon Nanodot Composite Hydrogel retained its efficient adsorption capacity, indicating its potential for recurrent application in the removal of Cr(VI) [75]. ​

These findings suggest that chitosan-based hydrogels can be effectively regenerated and reused for Cr(VI) adsorption, maintaining high removal efficiencies over multiple cycles. The proper selection of desorping agents and conditions plays a significant role in the regeneration process, with alkaline solutions like NaOH commonly used to desorb Cr(VI) from the hydrogel matrix. Optimizing these conditions is very important to guarantee minimal degradation of the hydrogel structure and sustained adsorption performance.

### Proposed mechanisms for Cr(VI) adsorption onto the prepared sorbents

**Fig. 7:**
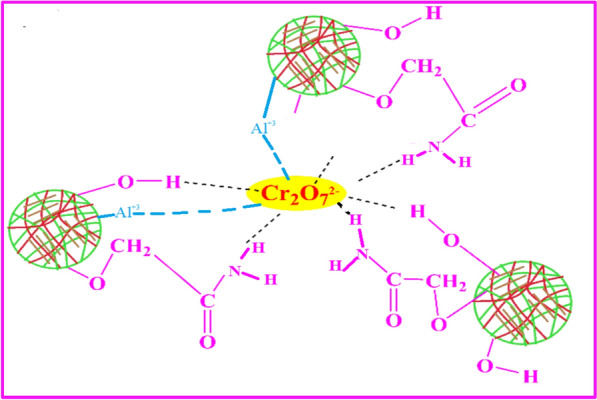
Proposed mechanisms for Cr(VI) attachment to the prepared chitosan-based sorbents

As per our previous work [27], it is assumed that the interaction of Cr(VI) onto the prepared sorbents may be achieved via multiple mechanisms that contribute to the overall removal efficiency (Fig. 7). The chief mechanisms include electrostatic attraction, reduction, and chelation/complexation as following: ​

1. Electrostatic Attraction: In acidic environments, the abundant amino groups (-NH₂) existing on chitosan become protonated to form -NH₃⁺, making the hydrogel positively charged. This positively charged surface readily attracted to the negatively charged Cr(VI) species, such as HCrO₄⁻ and Cr₂O₇^2^⁻, leading to their adsorption onto the hydrogel matrix. The competence of this process is highly pH-dependent, with optimal adsorption typically occurring under acidic conditions (pH < 3) [12]. ​
2. Reduction of Cr(VI) to Cr(III) (at neutral of alkaline pH): Chitosan possesses reducing properties (due to the electron donating groups such as –NH₂ and O–H) that turn the more toxic Cr(VI) to trivalent chromium [Cr(III)] which is less toxic and more stable. This species can complexate with functional groups on the hydrogel or precipitate as Cr(OH)₃ under appropriate pH conditions. ​
3. Chelation/Complexation: Electron donating groups within the chitosan-based hydrogel, such as hydroxyl (−OH) and amino (-NH₂) groups, can readily interact with Cr(III) ions to form stable chelates or complexes. This mechanism enhances the retention of chromium species within the hydrogel network, contributing to the overall adsorption capacity. ​These mechanisms often work synergistically, with the specific contributions depending on other structural factors such as the stability of the chelate structure, degree of deacetylation of chitosan, existing of other functionalities, type of chemical modification. The conditions of the medium such as temperature, and the presence of other ions in solution also affect the overall adsorption mechanisms. Understanding these interactions is crucial for optimizing the design and application of chitosan-based hydrogels for effective Cr(VI) remediation. The hydrogels investigated in this work contain aluminum oxide as an inorganic support. In this regard, chitosan-metal oxide composites were a topic of interest for their efficacy in removing hexavalent chromium [Cr(VI)] from aqueous solutions. These composites leverage the synergistic effects of chitosan's biocompatibility and metal oxides' adsorption capabilities. For instance, Dinh et al. studied Chitosan-MnO₂ Nanocomposite which demonstrated a Langmuir monolayer adsorption capacity of 61.56 mg/g for Cr(VI), indicating its potential for effective removal of chromium from water [23].

In another work, Zhang et al. declared that Incorporating magnetite and graphene oxide into chitosan matrices to prepared Chitosan/Magnetite-Graphene Oxide Composite resulted in enhanced adsorption performance, with the composite achieving significant Cr(VI) removal under various conditions. These studies highlight the effectiveness of chitosan-metal oxide composites in Cr(VI) remediation, emphasizing the importance of composite composition, pH, and temperature in optimizing adsorption performance [76].

### A Comparison between the removal performance of the prepared sorbents and those mentioned in the previous works

A brief comparison is held to compare the removal performance of the claimed chitosan-based sorbent materials and those investigated by other researchers, the data are collected in Table (3). It can be seen that the claimed materials are effective candidates for removal of toxic Cr(VI) from aqueous media at very low sorbent dose, only 0.1 g. This finding together with the data of MR dye [4] confirm the suitability of the prepared sorbents for wastewater treatment applications (Table 3).

**Table 3:** Comparative effectiveness of Cr(VI) removal onto several sorbents based on chitosan

Formulations	Conditions	Q*_max_ mg/g	Refs.
Activated carbon from cassava peels	pH = 4, 10 mg initial Cr(IV) concentration, 55 °C	166.35	[15]
Carboxymethyl Chitosan‑Nanoclay	pH = 8, 90 min., 50 °C, 0.05 g adsorbent	205	[27]
Green Moringa leaves biomass	pH 2, 60 min.100 mg/l initial Cr(IV) concentration	33.9	[68]
Cs_1_/AAm_1_/Gly_1_/Al_0.05_	pH = 2, 0.1 g adsorbent, 180 min., 45 °C	51.5	This work
Cs_2_/AAm_2_/Gly_1_/Al_0.05_		50.9	
Cs_1_/AAm_1_/Gly_2_/Al_0.05_		50.43	
N-CMCs_1_/AAm_1_/Gly_1_/Al_0.05_		51.3	
N-CMCs_2_/AAm_2_/Gly_1_/Al_0.05_		48.9	
N-CMCs_1_/AAm_1_/Gly_2_/Al_0.05_		50.8	

Q*_max_, approximated to the nearest tenth

### Kinetic isotherms

**Fig. 8:**
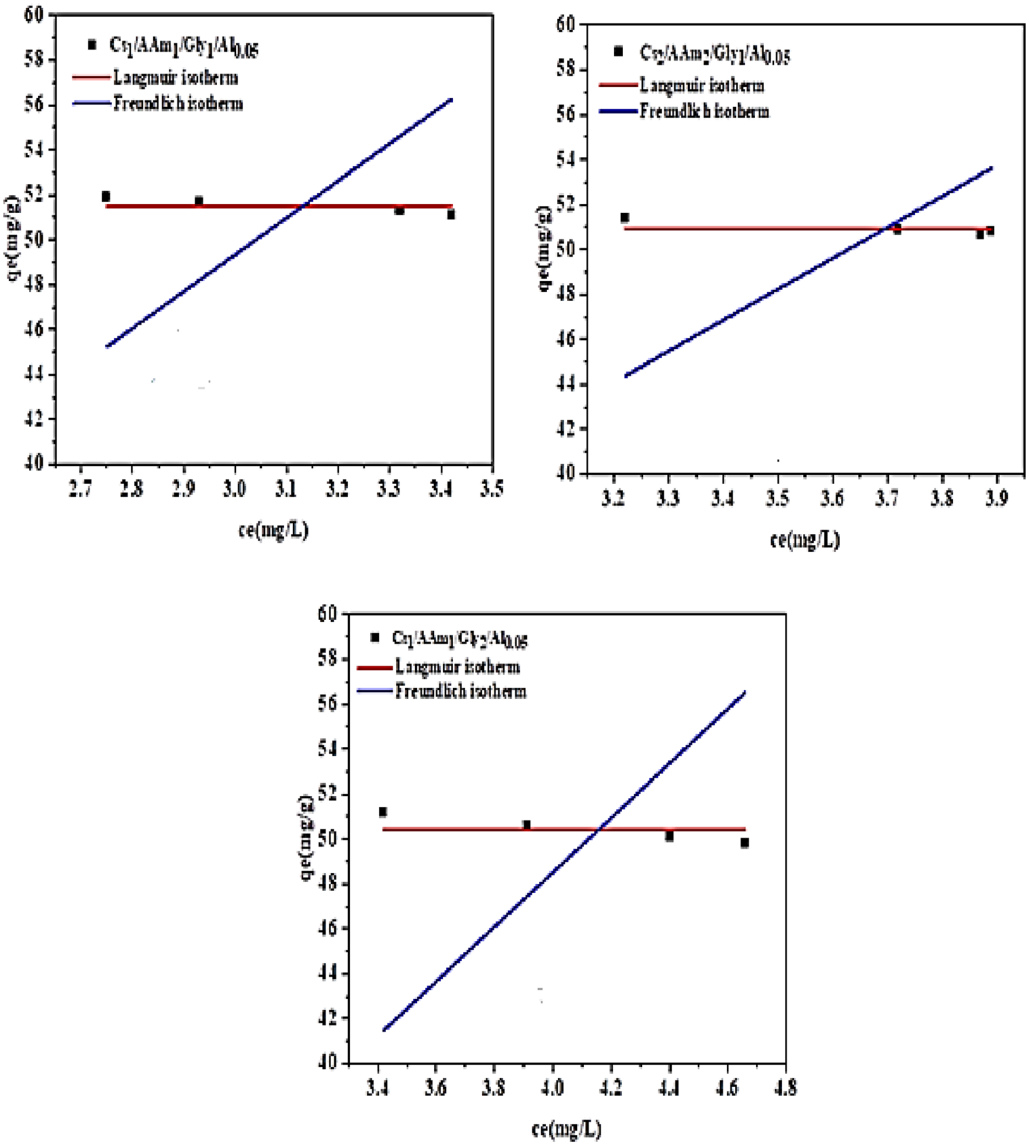

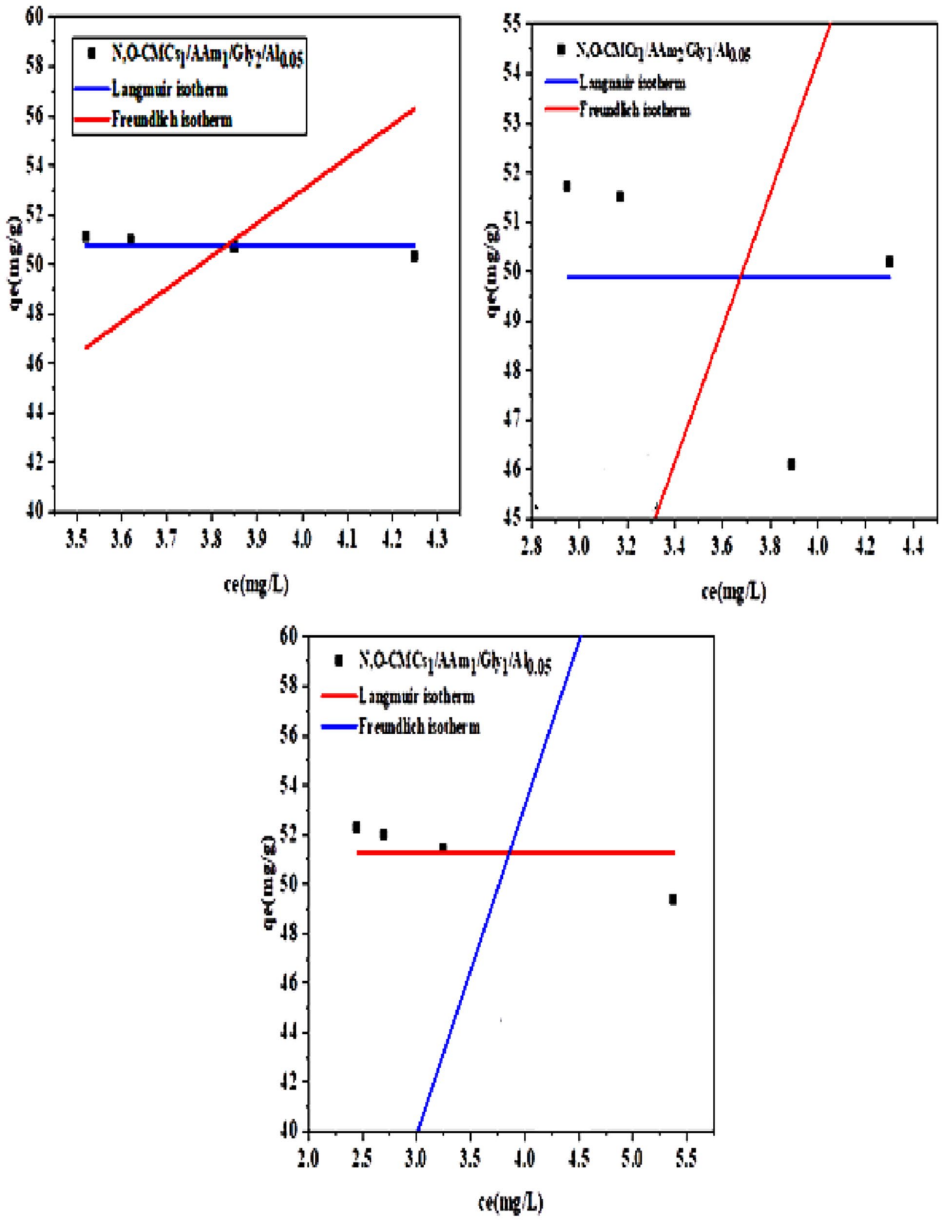
Kinetic isotherms **a** for elements of group A, **b** elements of group B

Two kinetic isotherms, Langmuir and Freundlich, were fitted in this study to examine the characteristics of the Cr (VI) adsorption process onto the produced chitosan-based sorbents. These are presented in Fig. 8a and b for the components of groups A and B, in turn. The Langmuir model states that when monolayer adsorption occurs on a uniformly homogenous surface, there is no contact between the adsorbent and the adsorbate. However, the Freundlich model participates in a multilayer adsorption process due to the interaction between the molecules of the adsorbate and the surface of the adsorbent. The adsorption data from Freundlich and Langmuir isotherms may be compared to identify the best-fitting isotherm model and the adsorption process mechanism. The information is provided in Table 4. It can be observed that R^2^ values for all the samples are obtained for the Freundlich model, which implies a multi-layer adsorption process. This might be because sorbents with different functional groups are more suited for multilayer adsorption [60]. It is important to note that all of the sorbents under investigation had respectable Q_max_ values, indicating that their removal capabilities were very comparable.

**Table 4:** Kinetic isotherm data

Langmuir: qe = (Qm*ka*ce)/(1 + (ka*ce))		Freundlich: qe = kf*(ce)^(1/nf)	
*Cs1/AAm1/Gly1/Al0.05*			
Q_m_	51.5	kf	12.6718
Ka	**0.09657**	nf	**1.16244**
R-Square (COD)	**0.95121**	R-Square (COD)	**0.99944**
*Cs*_*1*_*/AAm*_*2*_*/Gly*_*1*_*/Al*_*0.05*_			
Q_m_	50.9497	kf	12.1403
Ka	**0.09665**	nf	**1.15528**
R-Square (COD)	**0.96581**	R-Square (COD)	**0.99885**
*Cs*_*1*_*/AAm*_*1*_*/Gly*_*2*_*/Al*_*0.05*_			
Q_m_	50.425	kf	12.3193
Ka	**1.86204**	nf	**1.19123**
R-Square (COD)	**0.02569**	R-Square (COD)	**0.96443**
*N,O-CMCs*_*1*_*/AAm*_*1*_*/Gly*_*1*_*/Al*_*0.05*_			
Q_m_	51.275	kf	12.2117
Ka	**0.05143**	nf	**1.1665**
R-Square (COD)	**0.97699**	R-Square (COD)	**0.99014**
*N,O-CMCs2/AAm*_*2*_*/Gly*_*1*_*/Al*_*0.05*_			
Q_m_	49.8744	kf	12.1721
ka	0.08744	nf	**1.16001**
R-Square (COD)	0.93874	R-Square (COD)	**0.97065**
*N,O-CMCs*_*1*_*/AAm*_*1*_*/Gly*_*2*_*/Al*_*0.05*_			
Q_m_	50.775	kf	12.2161
Ka	**0.04605**	nf	**1.16726**
R-Square (COD)	**0.95332**	R-Square (COD)	**0.97465**

### Adsorption models

**Fig. 9:**
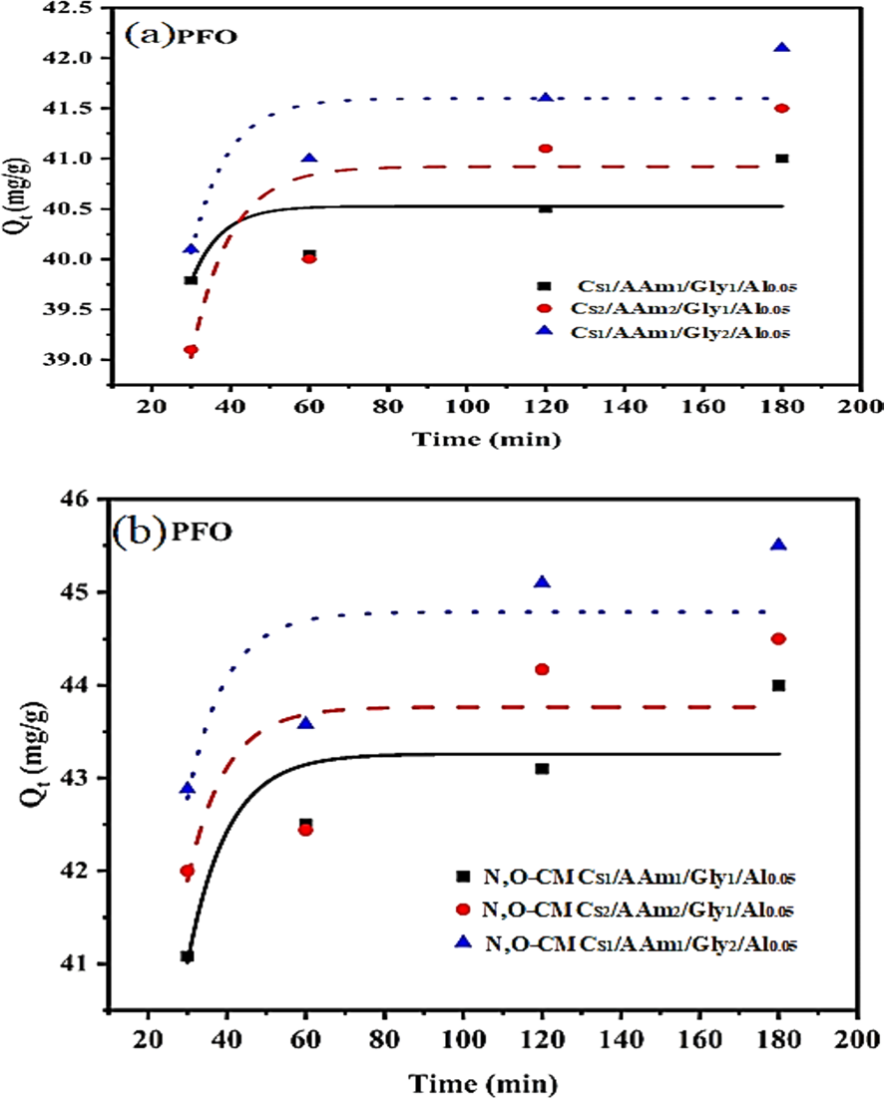
Pseudo-1st order kinetic model

**Fig. 10:**
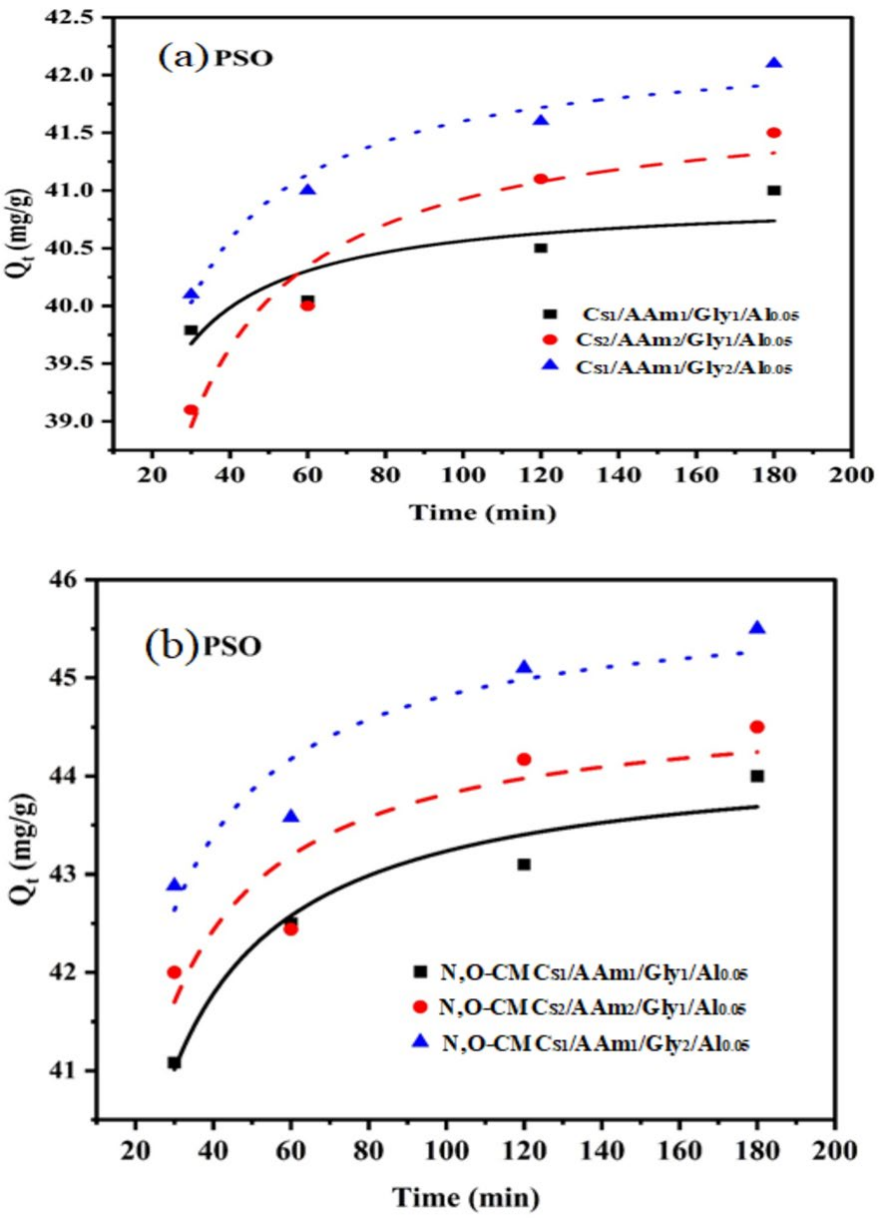
Pseudo-2nd order kinetic model

Pseudo-first and second-order models were verified to confirm the adsorption process's rate or order. They are presented in following non-linear equations:

7 $${q}_{t}={q}_{e}\left(1-{e}^{-{k}_{1}t}\right) \text{Pseudo}-\text{first order}$$

8 $${q}_{t}=\frac{{k}_{2}{q}_{e}^{2}t}{1+{k}_{2}{q}_{e}t} \text{Pseudo}-\text{second order }$$

where qt and qe are the concentrations of MB adsorbed at time t and equilibrium e, respectively, and k1 and k2 are the pseudo-1st-order and pseudo-2nd-order rate constants for the adsorption. Figures 9 and 10 display the data, respectively. Additionally, Table 5 provides the adsorption parameters. It can be observed that, in optimal conditions, the adsorption process is pseudo-second-order, meaning that the active groups on the sorbent molecules and the Cr(VI) chemisorb each other. Our findings align with those presented in the study on adsorption kinetic models by Emmanuel et al. [52]. Additionally, Al-Ghouti and Dana hypothesized that a pseudo-second-order model [6] accounted for the adsorption of Cr(VI) into some agrowastes.

**Table 5:** Data for kinetic models

Kinetic Model	Kinetic Parameters	Cs_1_/AAm_1_/Gly_1_/Al_0.05_	Cs_2_/AAm_2_/Gly_1_/Al_0.05_	Cs_1_/AAm_1_/Gly_2_/Al_0.05_
Pseudo -first order	K_1_ (min^−1^)	0.1328	0.10237	0.10991
PFO	qe (Cal.), (mg/g)	40.52	40.92	41.59
	R^2^	0.48202	0.69919	0.75386
	Chi square	0.21954	0.53356	0.27322
Pseudo -second order	K_2_ (g mg^−1^ min^−1^)	0.02513	0.01077	0.0138
PSO	qe (cal), (mg/g)	40.9	41.93	42.31
	R^2^	0.9999	0.9999	0.9999
	Chi square	0.08203	0.08492	0.03638
Kinetic Model	Kinetic Parameters	N,O-CMCs_1_/AAm_1_/Gly_1_/Al_0.05_	N,O-CMCs_2_/AAm_2_/Gly_1_/Al_0.05_	N,O-CMCs_1_/AAm_1_/Gly_2_/Al_0.05_
Pseudo -first order	K_1_ (min^−1^)	0.09858	0.10509	0.10348
PFO	qe (Cal.), (mg/g)	43.26083	43.7645	44.78923
	R^2^	0.78022	0.51007	0.5956
	Chi square	0.4957	1.13283	0.93213
Pseudo -second order	K_2_ (g mg^−1^ min^−1^)	0.0095	0.01003	0.00971
PSO	qe (cal), (mg/g)	44.26404	44.79216	45.8277
	R^2^	0.99998	0.99994	0.99996
	Chi square	0.10035	0.37732	0.24055

### Real adsorption experiment for an actual textile wastewater sample via the synthesized sorbents

Table 6 lists the experimental results for applying the optimized samples using the tea bag technique to a genuine textile wastewater sample after two hours. It is evident that the two optimized sorbents displayed exceptionally well performance in removing of Cr(V) in presence of other pollutants (Cu^+2^: 131 ppm, Cr^+6^: 33.8 ppm, Hg^+2^ = 21.17 ppm and Pb^+2^ = 14.18 ppm). This result guarantees that the investigated sorbents may be used as a practical solution for treating metal-containing dye effluents.

**Table 6:** Data for application on real textile wastewater sample

Sample	Concentration of pollutant (ppm)		Removal percentage (%)	
	*MR*	***Cr (VI)***	*MR*	***Cr (VI)***
Wastewater sample	338	33.8	–	–
Cs1/Aam1/Gly1/Al0.05	44	12.14	86.9	64
N, OCMCs1/Aam1/Gly1/Al0.05	31	9.88	90.8	70.8

### Selectivity investigation

**Fig. 11:**
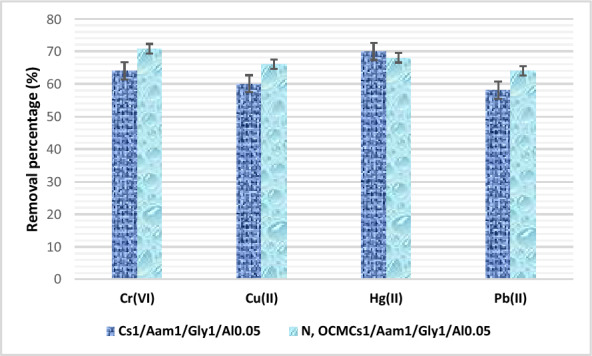
Selectivity of the prepared hydrogels towards some heavy metals

In order to elucidate the selectivity of the prepared sorbents towards Cr(VI), the concentration of other heavy metals existing in dye effluent was measured after treatment with the two optimized hydrogels. The data are presented in Fig. 11. It can be seen that the removal performance of the hydrogels towards Cu^+2^, Pb^+2^ and Hg^+2^ is relatively close to that proved towards Cr(VI) implying the suitability of the prepared hydrogels as effective candidate in wastewater remediation application. The preference of the sorbents towards a specific metal over the others may be attributed to the stability of the complex formed or relied on the overall metal-hydrogel binding mechanisms.

## Conclusions

This study systematically synthesized and evaluated six gamma-irradiated (30 kGy) chitosan-based hydrogel composites for efficient Cr(VI) removal from both synthetic aqueous solutions and real dye wastewater containing competing cations. Comprehensive structural characterization (FTIR, XRD, TGA)- given in first part of this study (Ref. 44) confirmed the successful combination of Al_2_O_3_ into the gel matrix for enhancing removal performance and exhibiting comparable adsorption capacity (> 50 mg/g). pH-dependent studies identified optimal Cr(VI) removal at pH 2 (94.3 ± 1.5% efficiency), attributable to enhanced protonation of active sites and different Cr(VI) forms existing at the investigated pH range. Kinetic analysis (R^2^) validated pseudo-second-order behavior, confirming chemisorption via electrostatic attraction and redox reactions. Isotherm modeling demonstrated Freundlich dominance (n = 2.1–3.4), reflecting multilayer adsorption on energetically heterogeneous surfaces. The hydrogels maintained > 85% removal efficiency after five regeneration cycles (0.1 M NaOH elution), underscoring their reusability. These findings highlight the efficacy of structural tailoring (carboxymethylation + Al_2_O_3_ enforcement + irradiation crosslinking) in developing sustainable chitosan sorbents. Given their cost-effectiveness (cheap raw materials with facile preparation protocol), environmental safety (non-toxic byproducts), and performance in complex matrices (real wastewater removal: 82.7 ± 2.1%), these composites present a scalable solution for industrial Cr(VI) remediation. Future work should prioritize pilot-scale validation and lifecycle assessment to bridge lab-to-industry.

## Data Availability

All the data are provided in the present manuscript.
